# Functional Trait Changes, Productivity Shifts and Vegetation Stability in Mountain Grasslands during a Short-Term Warming

**DOI:** 10.1371/journal.pone.0141899

**Published:** 2015-10-29

**Authors:** Haifa Debouk, Francesco de Bello, Maria-Teresa Sebastià

**Affiliations:** 1 Laboratory of functional ecology and global change (ECOFUN), Forest Sciences Centre of Catalonia (CTFC), Solsona, Spain; 2 Group GAMES and department of Horticulture, Botany and Gardening, School of Agrifood and Forestry Science and Engineering, University of Lleida, Lleida, Spain; 3 Department of Botany, Faculty of Sciences, University of South Bohemia, České Budějovice, Czech Republic; 4 Institute of Botany, Czech Academy of Sciences, Třeboň, Czech Republic; WSL Institute for Snow and Avalanche Research SLF, SWITZERLAND

## Abstract

Plant functional traits underlie vegetation responses to environmental changes such as global warming, and consequently influence ecosystem processes. While most of the existing studies focus on the effect of warming only on species diversity and productivity, we further investigated (i) how the structure of community plant functional traits in temperate grasslands respond to experimental warming, and (ii) whether species and functional diversity contribute to a greater stability of grasslands, in terms of vegetation composition and productivity. Intact vegetation turves were extracted from temperate subalpine grassland (highland) in the Eastern Pyrenees and transplanted into a warm continental, experimental site in Lleida, in Western Catalonia (lowland). The impacts of simulated warming on plant production and diversity, functional trait structure, and vegetation compositional stability were assessed. We observed an increase in biomass and a reduction in species and functional diversity under short-term warming. The functional structure of the grassland communities changed significantly, in terms of functional diversity and community-weighted means (CWM) for several traits. Acquisitive and fast-growing species with higher SLA, early flowering, erect growth habit, and rhizomatous strategy became dominant in the lowland. Productivity was significantly positively related to species, and to a lower extent, functional diversity, but productivity and stability after warming were more dependent on trait composition (CWM) than on diversity. The turves with more acquisitive species before warming changed less in composition after warming. Results suggest that (i) the short-term warming can lead to the dominance of acquisitive fast growing species over conservative species, thus reducing species richness, and (ii) the functional traits structure in grassland communities had a greater influence on the productivity and stability of the community under short-term warming, compared to diversity effects. In summary, short-term climate warming can greatly alter vegetation functional structure and its relation to productivity.

## Introduction

High mountain ecosystems are considered to be particularly vulnerable to global warming [1,2]. Under climate change conditions, floristic biodiversity in European semi-natural, species-rich and subalpine grasslands is expected to be highly affected [3–5]. A number of studies have observed that warming enhances productivity [6–10], alters species composition and reduces species diversity [10,11], at least in a short term. The increase in biomass productivity has been attributed to the enhanced plant growth as a transient result of increased soil organic matter mineralization [12,13] and nutrient availability with warming [6,8]. These resource enriched environmental conditions are considered favorable to species characterized by fast growth and high returns on resources investment [14,15]. This means that conservative species, with slower nutrient acquisition and slower growth (as opposed to acquisitive species, Diaz et al. [16]) could be out-competed as a result of nutrient depletion by fast-growing species [17] or could disappear as a result of low adaptation ability to the new conditions. Increased productivity can, therefore, either increase or reduce diversity depending on the relationship between productivity and diversity [18,19].

The impact of warming should also depend on the resistance and resilience, altogether defining stability, of plant communities. In many studies, the stability of the community has been related to diversity—with an a priori known as the “diversity begets stability” hypothesis. The theory suggests a positive stability-diversity relationship because the system could be more stable when more species are available, thus providing a sort of insurance towards different environmental changes [20–23]. However, other studies question this hypothesis [24–26] because ecosystem stability could simply depend on the type of dominant species in the system. These studies advocate that the positive correlation between stability and diversity can be explained by the fact that higher diversity simply increases the probability of including species with traits better adapted to the new environmental conditions [27–29]. They also argue that looking only at species composition and diversity does not give a mechanistic view on the ecosystem and does not allow generalizations beyond specific sites.

While several studies have assessed the effect of global warming on species diversity [3–5,30,31], the response of the community plant functional traits to increasing temperatures and their role in the vegetation stability have been generally overlooked. The importance of plant functional traits (any characteristics of organisms which impact its fitness) lies in the fact that they consistently reveal community responses to environmental changes across regions and control ecosystem processes (vegetative growth, photosynthesis, decomposition, etc. [32]). Species with distinct functional traits are expected to respond differently to environmental conditions, and hence influence ecosystem processes accordingly [33]. In warming experiments, it remains unclear how diversity and plant functional traits influence the community resistance and to which extent they contribute to its resilience under warming. It is also unclear how ecosystem processes such as productivity and stability will be affected by changes in vegetation structure and what type of species will become more dominant. Therefore, we expect that the study of functional traits can improve our understanding of the mechanisms related to the stability of the ecosystem, under warming.

In this study, we investigated the effect of warming on productivity, plant traits composition and functional diversity during one growing season through transplanting grassland turves from the highland (humid and cold) to the lowland (dry and warm) areas. We focus on semi-natural subalpine grasslands in the Eastern Pyrenees, which are considered to be vulnerable to climate change due to their position at the south-western edge of the semi-natural grassland biome in Europe [8]. Additionally, climate change scenarios in the region predict an increase in mean annual temperature of 0.5°C and a decrease in mean annual precipitation of 10% [34]. Particularly in the Spanish Pyrenees, effects of warming are believed to be particularly pronounced [35,36]. Many studies suggest that biodiversity enhances ecosystem function [37,38] and is indispensable for the stability of plant communities in terms of productivity and vegetation composition [20–22]. However stability does not only depend on species richness but also on the functional traits of the species and on the growth-related traits [25,26]. Therefore, we hypothesize that community traits associated to resource acquisition and competitiveness (specific leaf area, leaf dry matter content, start of first flowering, plant height, growth habit, rhizome-formation, etc.), together with species richness, will contribute to the stability of the community in the face of warming, in terms of vegetation composition. We also hypothesize that the increased productivity in the lowland is not exclusively the result of the initial species richness, but also of the functional trait changes under warming conditions. In our study, we analyze (1) the resistance of the grassland community to warming, (2) the effect of diversity and functional traits on biomass production, (3) and the effect of species and functional diversity and functional traits composition on community stability in terms of vegetation composition.

## Materials and Methods

### Transplanting

A short-term (one growing season) climate change experiment was carried out through transplanting grassland turves from highland (moist and cold) to lowland (dry and warm) areas. The highland study system consisted of two cold temperate semi-natural subalpine grassland sites (p1 and p2) at around 2000 m a.s.l. with a distance of approximately 1 km from one another at the Pla de Rus in Cadí-Moixerò Natural Park (1·993°E, 42·276°N). These subalpine grasslands are characterized by cold temperate climate (mean annual temperature is 5.3°C and mean annual precipitation is 1183.4 mm; Atlas Climàtic Digital de Catalunya http://www.opengis.uab.cat/acdc/, accessed in July 2013). The vegetation was dominated, in both sites, by subalpine perennial mesic grasslands on limestone dominated by *Festuca nigrescens* Lam., in addition to *Carex caryophyllea* Latourr., *Anthoxanthum odoratum* L., *Potentilla neumanniana* Reichenb., *Galium verum* L., *Thymus pulegioides* L., and *Koeleria macrantha* (Ledeb.) Schultes (see Sebastiá et al. [7] for more details on original vegetation and environment).

The experiment addressed mostly a temperature and water change scenario (see below) during the vegetative period of the plants, representing an extreme warming event. At each of the two highland sites, 60 turves were extracted and each turf was put in plastic trays of 40 cm x 40 cm x 20 cm (depth). Half of the turves from each site were selected randomly and placed back in the grassland (‘highland’ treatment). The other half was placed in experimental fields of the School of Agrifood and Forestry Science and Engineering of the University of Lleida (‘lowland’). The lowland site has a continental Mediterranean climate (mean annual temperature is 14.5°C and mean annual precipitation is 442 mm). Since the lowland site (Lleida) is exposed to very dry and warm conditions during summer, water was added weekly to approximate half of the natural rain frequency at the subalpine site and to prevent the mortality of the plants due to the high discrepancy in precipitation between the highland and the lowland. The short-term duration of the experiment implies that new colonizers did not have time to colonize the turves, and thus observed responses correspond only to internal responses of the original system. It should be noted that higher temperature and reduced water availability were the main stressors in the lowland compared to the highland. However, other factors could also differ between the highland and the lowland. Since we did not observe any obvious difference in pests and parasites between highland and lowland, we can safely attribute the observed changes to increased temperature (see published work on this data for a more detailed discussion; [8,11]). As revealed by this work, changes in mineralization could explain some of the patterns observed, but again these changes were triggered by the increase in temperature. The turves were randomly distributed within the lowland, but kept separated between two sites as in the highland. A 20 cm x 20 cm quadrat was fixed in the centre of the 60 turves. This quadrat was divided into a grid of 100 subquadrats of 2 cm x 2 cm each. All the species present in each quadrat were recorded every two weeks. Frequency records and species counts were taken seven times throughout the growing season in the highland and eight times in the lowland (for more detailed information about the experiment and vegetation composition changes see Sebastià 2007; Sebastià et al. 2008). We did only seven measurements at the highland site because the seventh measurement had already covered the end of the growing season in the subalpine grassland.

At the end of the experiment, 17 weeks after the start, above-ground biomass was estimated by harvesting vegetation on a 20 x 20 cm quadrat placed in the middle of a subset of 28 out of the 60 turves used for species frequency. A previous harvest was carried out in 28 different turves 10 weeks after the start of the experiment. The biomass data at the two dates were used to analyze the diversity-productivity relationship.

#### Ethics statement

This study did not involve protected or endangered species and it was not necessary to obtain specific permission to sample at any of the sampling sites.

### Community functional trait structure

We considered various relevant functional traits that are linked to different species responses to biotic and abiotic factors. These were: specific leaf area (SLA, leaf area per unit dry mass), leaf dry matter content (LDMC, the ratio of leaf dry mass to fresh mass), vegetative reproduction (presence of rhizomes or stolons), plant growth habit (erect vs. prostrate), mean plant height (H_mean_), and start of first flowering (month). Trait values of the species present were obtained from two traitbases, i.e. the LEDA traitbase [39], and the CLO-PLA traitbase [40] and complemented by specific standardized measurements for SLA [41] in the field. Only few trait values were acquired from de Bello et al. [42]. One important factor in the response of species to warming could be trait plasticity [43] which is considered one of the major means by which species can cope with new environmental conditions [44]. Unfortunately, we could not measure the traits directly in the field. As such, our analyses might only partially capture the full extent of plant community response to warming.

Two indices were considered to reveal the major characteristics of the community functional trait structure [45]. The first one is the community weighted means of the traits (CWM). The CWM for each trait is calculated as the mean of the trait values in the community, weighted by the relative abundance of each species [46]. For categorical traits, this corresponds to the relative abundance of species bearing a certain type of traits. The second index is a commonly used measure of functional diversity expressed with the Rao quadratic entropy [45]. The Rao index expresses the sum of trait dissimilarity between each pair of species in a turf and it is weighted by species relative abundance. This index is mostly independent from the number of species. It was expressed as equivalent numbers [47] and for all traits combined. The numbers equivalent of a diversity index indicates the effective diversity, i.e. how many effectively different species in terms of abundance and, in case of traits, functional difference [48], thus providing a more intuitive concept of diversity comparable to species richness. Using Rao not in equivalent numbers produced strongly related results (as they are strongly related mathematically). Two additional indices of species diversity were calculated: species richness and Simpson’s diversity, also expressed in terms of equivalent numbers [47].

### Data analysis

The effects of the short-term climate change on the CWM of the traits (SLA, % of rhizomes, % of prostrate, start of first flowering, LDMC, and mean plant height) and the diversity indices (species richness, Simpson, and Rao) were analyzed using repeated measures ANOVA; where transplant (highland and lowland), extraction site (p1 and p2 in highland) and time (as quantitative value, 1 to 7 or 8) are the fixed factors. Results by Sebastià [8] suggested an increase in above-ground biomass in the lowland in comparison to the highland. Therefore, we conducted multiple regression models to examine the effect of each of the diversity indices and CWM on above-ground biomass in the two sites in both the highland and the lowland. In all the multiple regression analyses, we selected the best model based on the Akaike information criterion [49] using stepwise regressions. Correlation tests between the traits and the diversity indices were carried out prior to including them in the regression models to minimize collinearity problems (S1 and S2 Tables). The stronger correlation was between species richness and Simpson’s diversity index (R = 0.864, S2 Table) but we decided to keep both variables because they cover different ecological implications.

Changes in vegetation composition between turves of the two treatments (the highland and the lowland) were already observed by Sebastià et al. [11], and a higher dissimilarity in species composition was detected among the highland samples in comparison to the lowland, suggesting a change in the vegetation composition with warming. To determine the dynamics of change in vegetation composition within the turves as a result of the transplant, we calculated the Bray-Curtis dissimilarity [50] at the first and the second harvest (end of experiment) within each turve, using the CRAN package “vegan” in R software [51]. Multiple regression models were carried out to test the effect of each of the initial diversity indices and the initial CWM of traits on the change in vegetation composition (Bray-Curtis) in both the highland and the lowland (explanatory variables). We made additionally a stepwise regression model including all the variables of diversity and CWM traits with the Bray-Curtis dissimilarity. All statistical analyses were carried out using R software [52].

## Results

### Functional structure

**Fig 1:**
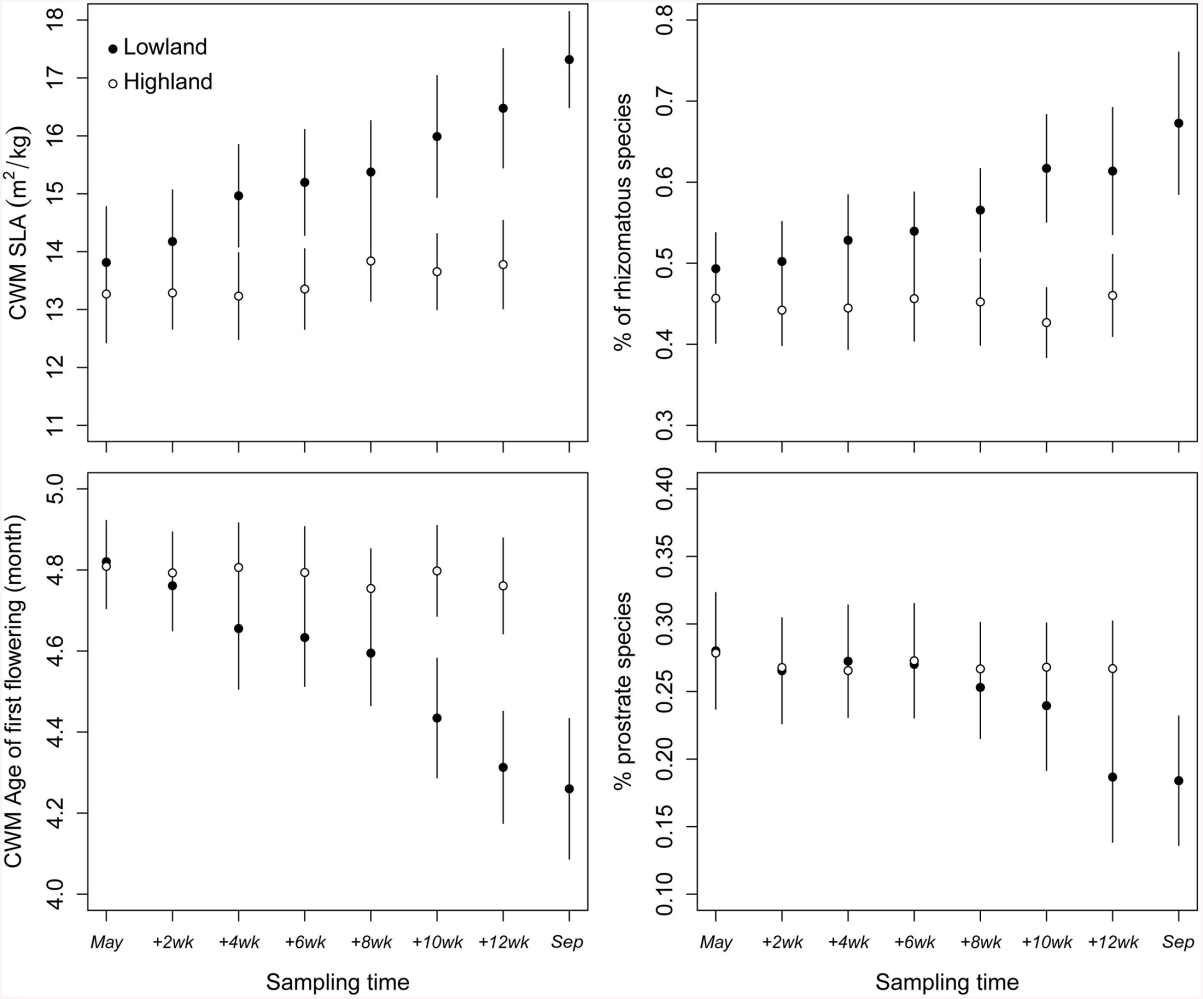
Effect of the transplant experiment on the CWM of traits. Effect of the transplant experiment on the Community weighted means (CWM) of SLA (upper left), % of rhizomatous species (upper right), start of first flowering (lower left), and % of prostrate plants (lower right) along time. The black points correspond to the lowland, and the white points correspond to the highland. The whiskers refer to standard deviation. The x axis indicates the dates of the repeated samplings (frequency) within each turf. The first sampling was done in mid May and the last one in the lowland at the beginning of September. For the corresponding statistical tests see Table 1.

**Fig 2:**
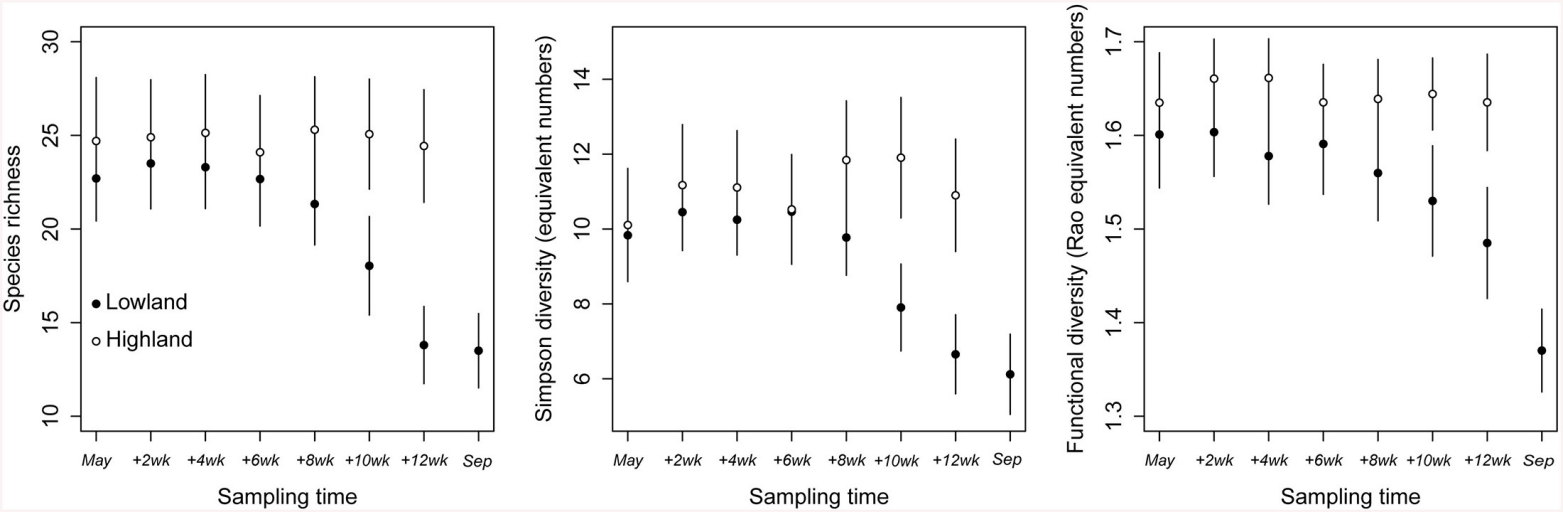
Effect of the transplant on the diversity indices. Effect of the transplant experiment on diversity indices: Species richness (left), Simpson’s reciprocal diversity index with equivalent numbers (centre), and Functional diversity’s index Rao with equivalent numbers (left) along time. The black points correspond to the lowland, and the white points correspond to the highland. The whiskers refer to standard deviation. See Fig 1 for more information on sampling dates and Table 1 for the corresponding statistical tests.

After the transplant, the functional structure of the grassland communities changed considerably (Table 1; Figs 1 and 2). Few weeks after the transplant, the community weighted mean (CWM) of SLA, start of first flowering and proportion of rhizomatous and prostrate species started to diverge from the lowland to the highland. The functional structure in the highland remained relatively unchanged along the growing season (Fig 1). Communities in the lowland appeared to be progressively more dominated by species with higher SLA, earlier flowering, erect growth habit and with rhizomes (Table 1; Fig 1). However leaf dry matter content (LDMC) did not respond to the short-term warming (Table 1). All those responses occurred based on the original species composition in each turf (we observed no newcomer appearing in the turves). Such an increase in abundance by a certain type of species was accompanied by the disappearance of other species (lower species richness and Simpson diversity) in the lowland (Fig 2). These two diversity components, in addition to functional diversity, decreased significantly in magnitude after the transplant (Fig 2; Table 1).

**Table 1:** Results of repeated measures ANOVA to assess the effect of the transplant on diversity indices and CWM traits with transplant (highland and lowland), site (p1 and p2 in the highland) and time. See Figs 1 & 2 for a graphical representation.

	Transplant		Site		Time		Transp. x Time		Site x Time		Transp. x Site x Time	
	F	*P*	F	*P*	F	*P*	F	*P*	F	*P*	F	*P*
***Diversity***												
**Species richness**	25.502	<0.001	0.791	0.3776	156.37	<0.001	126.002	<0.001	5.613	0.0183	6.455	**0.0115**
**Simpson**	14.113	<0.001	0.041	0.8396	60.595	<0.001	134.248	<0.001	0.462	0.4971	18.931	**<0.001**
**Rao**	22.137	<0.001	0.119	0.7312	87.449	<0.001	55.558	**<0.001** ^a^	2.159	0.1426	1.473	0.2256
***CWM traits***												
**SLA**	32.091	<0.001	0.014	0.906	142.03	<0.001	52.828	**<0.001**	1.483	0.224	0.031	0.859
**% Rhizomes**	27.521	<0.001	7.630	0.008	60.605	<0.001	53.480	**<0.001**	0.014	0.907	1.570	0.211
**% Prostrate**	1.426	0.238	1.264	0.266	47.863	<0.001	27.925	<0.001	8.599	0.004	4.681	**0.031**
**Start of first flowering**	17.462	<0.001	2.464	0.122	132.52	<0.001	80.168	**<0.001**	3.52	0.061	0.659	0.417
**LDMC**	1.079	0.303	0.005	0.942	4.211	**0.041**	3.428	0.065	0.42	0.517	0.033	0.856
**Mean Height**	0.03	0.863	0.901	0.347	4.648	0.032	1.134	0.288	1.069	0.302	9.826	**0.002**

^a^P-values in bold indicate significant relationships.

**Fig 3:**
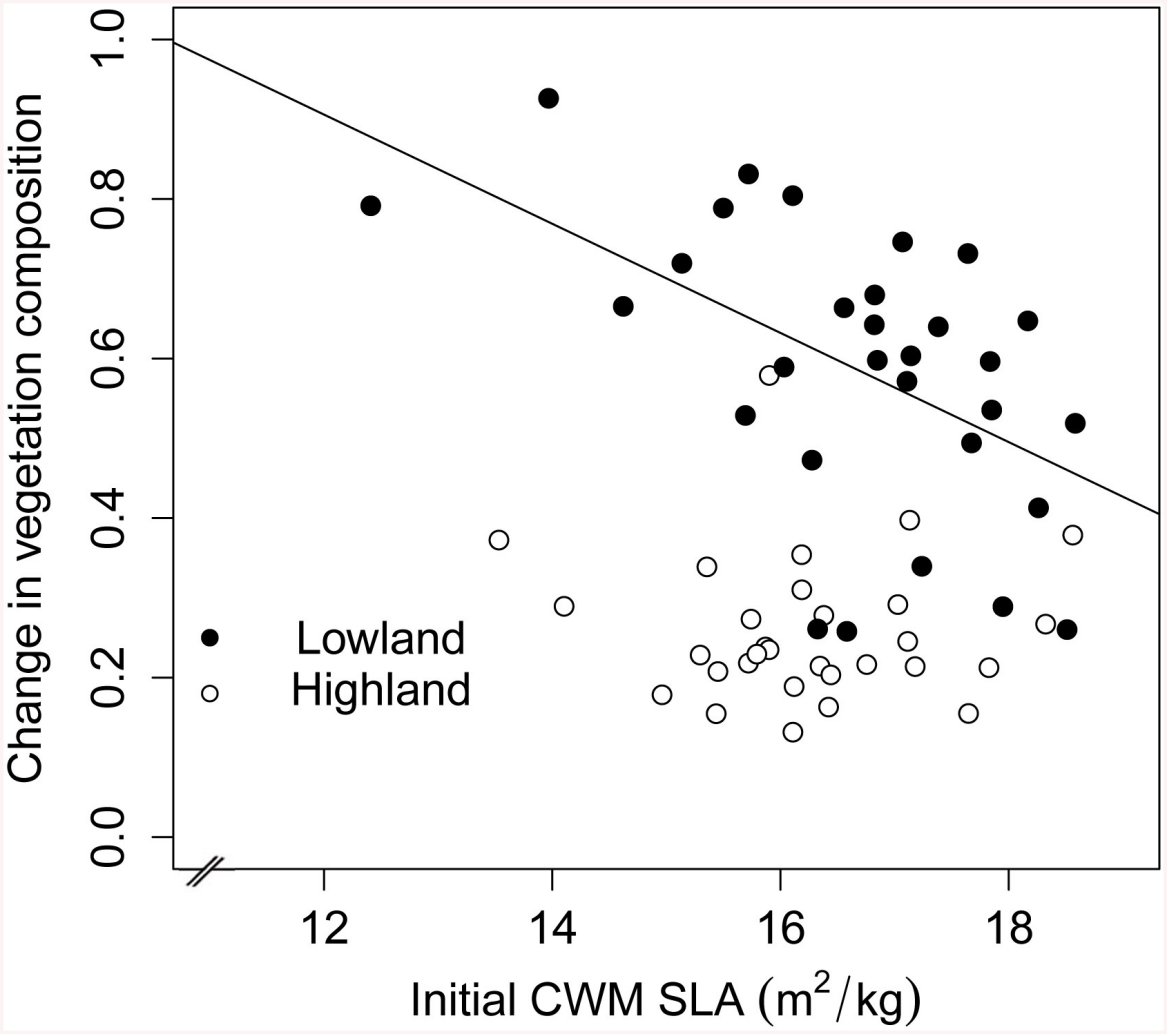
Relationship between the initial CWM SLA and the change in vegetation composition. Relationship between the initial CWM SLA and the change in vegetation composition (Bray-Curtis dissimilarity) between beginning and end of experiment, in both the highland and the lowland. The black points correspond to the lowland, and the white points correspond to the highland. Solid linear regression line reflects significant relationship (p < 0.05) in the lowland; and no line represents no significance.

We tested the change in vegetation composition within turves in both the highland and the lowland with multiple regression models and found that neither the initial diversity nor the initial functional traits diversity, had an influence on the change in vegetation composition (Table 2 and S3 Table). On the contrary the communities with initially low CWM of SLA witnessed a significant greater change in vegetation composition after the transplant (Fig 3; S3 and S5 Tables). Our results show that, in the lowland, turves originally dominated by species with higher SLA were more stable as they did not change considerably in terms of vegetation composition under warming. On the other hand, in plots originally dominated by species with lower SLA, species characterized with high SLA replaced those with low SLA as a result of warming (Fig 3).

**Table 2:** Results of multiple regression models to assess the effect of the diversity indices (species richness SR, Simpson’s diversity index, functional diversity FD) on above-ground biomass (final harvest) and changes in vegetation composition (Bray-Curtis), with transplant (highland and lowland) and site (p1 and p2 sites in the highland). See Fig 4 for a graphical representation of the biomass models.

	Above-ground biomass			Bray-Curtis		
	Model	R^2^_adj._	*P*	Model	R^2^_adj._	*P*
	***^a^	0.810		***	0.538	
**Site**			0.741			0.568
**Transplant**			0.989			0.102
**SR**			0.144			0.932
SR*transplant			**0.008** ^b^			0.852
	***	0.690		***	0.543	
**Site**			0.829			0.325
**Transplant**			0.803			0.406
**Simpson**			0.636			0.414
Simpson*transplant			0.405			0.570
	***	0.800		***	0.540	
**Site**			0.390			0.441
**Transplant**			0.328			0.592
**FD**			0.067			0.682
FD*transplant			**0.008**			0.685

^a^***P < 0.001

^b^P-values in bold indicate significant relationships.

### Productivity

**Fig 4:**
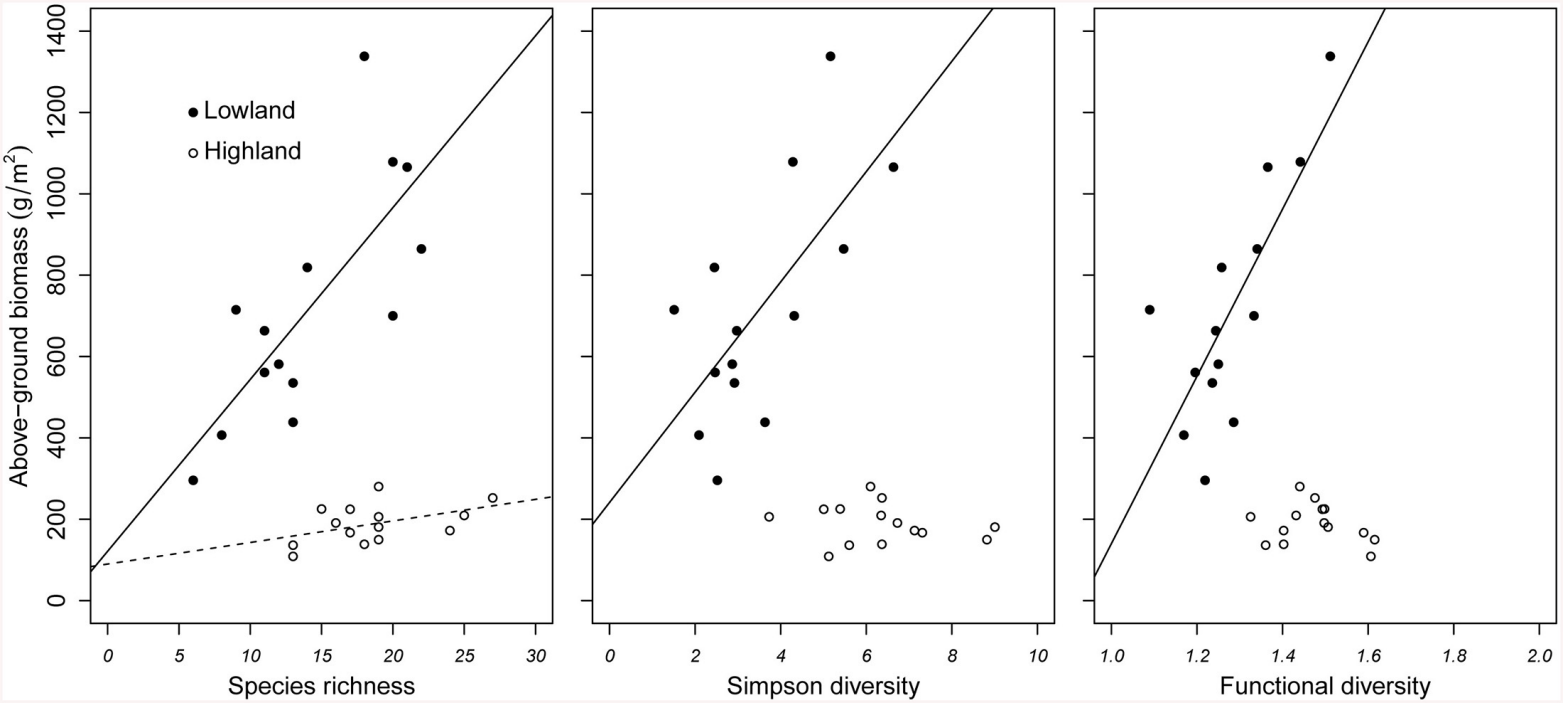
Relation between above-ground biomass and diversity indices. Relation between above-ground biomass and diversity indices: Species richness (left), Simpson’s diversity index with equivalent numbers (centre), and Functional diversity’s index Rao with equivalent numbers (right) in both the highland and the lowland. The black points correspond to the lowland, and the white points correspond to the highland. Solid linear regression line reflects significant relationship (p<0.05) between the variables in the lowland; dashed line refers to a marginal significant correlation (p<0.1) in the highland; and no line represents no significance.

We observed that the productivity in the grassland community under warming increased with diversity (Fig 4; Table 2), particularly in the last harvest. The results of the best multiple regression model showed a significant positive effect of diversity on biomass, except for Simpson’s diversity index (Table 2).

Plant functional trait composition (CWM) also seems to affect productivity, either negatively or positively (Table 3). Particularly, in the lowland, biomass production increased in communities with taller and less rhizomatous species (*estimate = 44*.*85* for height, *estimate = -1091*.*43* for rhizomatous propagation, S4 Table). Whereas in the highland, biomass increased with more rhizomatous species (*estimate = 136*.*38*, S4 Table). Other traits, such as SLA and prostrate growth presented a significant positive relationship with biomass, in both the highland and the lowland (*estimate = 71*.*6*, *P* < 0.001 for SLA and *estimate = 405*.*66*, *P* < 0.05 for prostrate growth form, Table 3). Our stepwise regression results also showed that the CWM traits, particularly the height and the proportion of rhizomes, have a greater effect on above-ground biomass than diversity (S4 Table).

**Table 3:** Results of multiple regression models to assess the effect of the plant functional traits (SLA, LDMC, height, start of first flowering, % prostrate, % rhizomes) on above-ground biomass, with transplant (highland and lowland) and site (p1 and p2 sites in the highland).

	Above-ground biomass					
	Model	R^2^_adj._	Estimate	Std. Error	t value	*P*
**Site**	***^a^	0.984	-181.98	28.54	-6.38	**<0.001** ^b^
**Transplant**			-1683.23	426.60	-3.95	0.001
**LDMC**			1.49	0.88	1.69	0.111
**SLA**			71.60	13.48	5.31	**<0.001**
**Prostrate**			405.66	181.42	2.24	**0.040**
**Rhizomes**			2653.65	265.88	9.98	<0.001
**Height**			-16.46	10.87	-1.51	0.150
**Start of first flowering**			-995.52	193.78	-5.14	<0.001
Transplant*rhizomes			-2156.80	175.02	-12.32	**<0.001**
Transplant*height			21.37	9.34	2.29	**0.036**
Transplant*start of first flowering			693.08	137.38	5.05	**<0.001**

^a^***P < 0.001

^b^P-values in bold indicate significant relationships (P-values < 0.05)

## Discussion

We found that short-term climate warming can have a great impact on the functional structure of grassland communities, both in terms of CWM of traits (Fig 1; Table 1) and functional diversity (Fig 2; Table 1). No major changes in the highland were observed along the growing season in terms of CWM of traits and diversity, suggesting that changes in the lowland are related to climatic stress and not seasonality. The significant interaction “transplant x site x time” suggests indeed that the pattern over time is different depending on which highland site the sample came from. While in some cases this interaction was significant, the estimates in Table 1 were often not very high and from visual inspection these effects were not strong. We therefore concluded that these effects, although existing were not very marked. Diversity variables and community traits showed different resistance in front of the new environmental conditions in the lowland (Figs 1 and 2). CWM of SLA and start of first flowering started shifting already after only two weeks whereas most of the other variables were resistant for around six weeks (Figs 1 and 2). Particularly, the proportion of prostrate species manifested a considerable resistance until the eighth week of the experiment before declining (Fig 1). Our results also showed that traits which provide opportunistic use of increased resource availability (in our case species with higher SLA, early flowering, erect growth habit, and rhizomatous strategy) became dominant as a result of the short-term climate change, while conservative species became less abundant or disappeared from the system (Fig 1; Table 1). Species with higher SLA, but not diversity components (species and functional diversity), contributed to the stability of the grassland community under warming conditions (Fig 3; Table 2 and S3 Table). In both the highland and the lowland, there was a positive relationship between species richness and productivity. However, in the lowland the vegetation was more productive and less diverse (as found by Sebastià et al. [11]). Overall the results suggest that plant functional traits, in terms of CWM, are the main drivers to the increased productivity in the lowland. We discuss these hypotheses and the patterns detected in the following sections.

### Community changes

Our results suggest that the warming in the lowland favored opportunistic erect species with higher SLA, anticipated flowering period, and more rhizomes (Fig 1). It is widely acknowledged that species from nutrient-rich habitats have a greater relative growth rate, hence greater SLA, manifested by fast resource capture and fast turnover of organs [53–55]. Mountain areas are characterized by low nutrient availability, where mineralization is often limited by cold temperatures [56,57]. Removing the temperature limitation in the lowland caused an increase in fast growth and resource acquisition species (e.g. higher SLA and earlier flowering). Earlier flowering of the plants as a response to warming was already observed in previous studies [58–60]. Despite increases of SLA as a response to short-term warming, it seems to be unresponsive to long-term climate warming [61]. The dominance of species with rhizomes under warming conditions (Fig 1) can be similarly explained by the fact that these species acquire the available nutrients faster compared with species that need to establish new stems [62]. Additionally, these species can store resources in the rhizomes from previous years (storage effect; Chesson [63]) and are therefore the fastest in reacting to improved environmental conditions (i.e. decrease in thermal constrain). Prostrate plants’ proportion decreased with time in the lowland compared to the highland (Fig 1). Increased abundance of erect plants in the lowland suggests that increased competition for light with enhanced biomass with warming [8,11] must have lead to the exclusion of prostrate plants [64].

Surprisingly, in spite of this increase of biomass and erect species proportion, plant height was not responsive to warming, and increased similarly in both locations throughout time. This was thus a plant growth effect, which likely increased competition for light during the growing season[65]. It could have also been the effect of using database traits, and not actual measurements. We also expected a change in LDMC, which is often related to SLA and that was reported to increase due to climate warming in Cantarel et al. [61]. However this variable did not change in our experiment, which could also be related to the high sensitivity of this trait to local conditions, not captured by database information. LDMC may vary considerably between different plants in the same site within the same day [41] and within the same species [66]. This variability could have been revealed if field measurements had been available. Nonetheless, for other traits mostly retrieved from databases, the observed patterns were however responsive, suggesting that trait information measured in other sites can provide valuable information on vegetation responses to environmental changes.

The shifts in CWM of traits were accompanied by a loss of species and a decrease in functional diversity, although our results show that the community-level traits (Fig 1) responded more rapidly to warming than species diversity (Fig 2). This was also observed by Suding et al. [67] and Cantarel et al. [61]. The reduction of species in the temporarily nutrient-rich habitat (lowland) may be the outcome of environmental filtering which excludes species that are less adapted to the warming period. It may also be the result of the out-competition of conservative species by opportunistic species with a faster growth rate (competitive exclusion principle, Gause [68]). The delay of the diversity indices in their response to warming until the eighth week of the experiment (Fig 2) indicates a certain resistance from the community to the simulated environmental conditions. This is possibly because of redundancy in the turves, followed by the disappearance of more conservative species. Opportunistic species are able to rapidly benefit from enhanced mineralization and exhibit higher growth rates and productivity relative to conservative species. Hence opportunistic species will cause the exclusion of weaker competitors. Indeed, we saw that opportunistic species, although not abundant before the transplant, became significantly dominant at the cost of more conservative species [11]. We underline that mountain subalpine grasslands consist of a wide range of opportunistic and conservative species with a high variety of survival-strategies. Our results suggest that grassland ecosystems may have the potential to cope well with new climate stress in the short term, thus being resistant to species invasion.

### Community stability

Our transplant experiment caused a shift in vegetation composition in the lowland favoring species with higher SLA under warming conditions. The biggest change in composition was observed where the turves had originally more species with lower SLA, and therefore species in these turves were replaced by others with greater SLA which acquired the resources more favorably under warming conditions (Fig 3; S3 and S5 Tables). On the contrary no diversity components (species and functional diversity) affected the stability of the grassland community under warming conditions. This shows that functional traits of the species (in our case SLA) exerted the major effect on the vegetation response, but not diversity. These results comply with previous findings highlighting the importance of plant functional traits and interspecific variability on population stability [43,69,70]. We thus conclude that the disappearance of species was greater in turves with originally lower SLA.

### Community stability-productivity

Sebastià [8] showed that above-ground biomass increased with warming in the lowland. Similar results were obtained by Lin et al. [9] and Baldwin et al. [10]. Cantarel et al. [61] confirmed this pattern in the short term but reported a progressive decline in the long term of the experiment. In our study, the above-ground biomass had a positive relationship with species richness within both the highland and the lowland (Table 3). This positive relationship between diversity and biomass production is based on Darwin's theory [71] and has been demonstrated in many studies [72–76]. These studies assume that nutrients are captured more efficiently in diverse plant communities because of species complementarity in their resource acquisition, and thus, as they suggest, a greater productivity is reached.

In our warming experiment, however, productivity increased and diversity decreased as a response to warming. Interestingly, plant traits and not diversity were the main factor to explain the increased productivity in the lowland. A greater biomass production was the outcome of faster mineralization rate and nutrient acquisition by opportunistic species that are taller, have greater SLA and erect form. Taller plants are considered more competitive since investment in height improves the access to light [77], allowing them to acquire nutrients faster thus leading to a faster growth and a greater productivity. The positive correlation between biomass and SLA was already described in Cornelissen et al. [41]. The positive relationship between biomass and prostrate plant growth may be caused by certain species such as *Koeleria macrantha* (33% prostrate; LEDA traitbase) which contributed greatly to the total biomass in each monolith [78], thus influencing significantly our results. This bias highlights the risk of using data from traitbases which may not be “optimal”. We conclude that the proportion of prostrate species in a community was tightly correlated in the biomass data with the presence of *K*. *macrantha*. We assume that on a long-term basis biomass production will decline, as previously seen in other warming experiments [61]. This decrease will occur when water and temporary flux of nutrients will get scarce with ongoing warming. Our short-term warming experiment favored the growth of opportunistic fast-growing species in the lowland. Communities dominated by fast-growing species have a higher resilience and a lower resistance to extreme events in comparison to slow-growing conservative species [25,79]. Therefore we expect that in the long-term, changes in resource availability and climate will lead to a new community at the expense of the old one [80]. We speculate that some species will acclimate through their functional traits to the long-term warming and drought and others will simply disappear if warm and dry years occur persistently as projected by climatic models for the region [81]. We also expect the invasion of the lowland by new species (annuals and woody species) more adapted to such extreme conditions, as suggested by the work of de Bello et al. [82–84].

## Conclusions

Overall, we found that a short-term warming enhanced productivity and reduced diversity significantly. A change in vegetation composition, manifested by a shift in dominance towards acquisitive fast-growing species, was also observed. Our results also advocate that a higher diversity did not contribute to a greater stability of the community under stress; instead plant functional traits, particularly high SLA, were responsible for the stability of the vegetation to increased temperature. Also, the changes in CWM of certain traits (% rhizomes, growth form, and start of first flowering) seem to be the main drivers of increased biomass production under climate warming. The relationship between productivity and species richness remained positive within both the highland and the lowland. We postulate that seasonal climate change strongly affects functional traits and diversity. On the long term, however, knowledge of sensitivity of grasslands to climate change is scant and thus more experiments over longer periods are needed. Particularly, complementary observational studies and reciprocal transplanting (from the lowland back to the highland) could be useful tools to better understand the observed patterns. Also, we suggest that further studies should address the recovery of the vegetation after a short period of climate warming, in terms of functional diversity and plant functional traits.

## Supporting Information

S1 Table Correlation between the CWM of traits. Correlation coefficients between the CWM of traits: SLA, LDMC, height, start of first flowering, % prostrate, % rhizomes.
(PDF)

S2 Table Correlation between the diversity indices. Correlation coefficients between the diversity indices: species richness SR, Simpson’s diversity index, and functional diversity FD.
(PDF)

S3 Table Relationship of initial plant functional traits with vegetation composition. Results of multiple regression models to assess the effect of the initial plant functional traits (SLA, LDMC, height, start of first flowering, % prostrate, % rhizomes) at the beginning of the experiment on vegetation composition (Bray-Curtis dissimilarity), with transplant and site. See Fig 3 for a graphical representation.
(PDF)

S4 Table Stepwise regression for the effect of CWM traits and diversity on biomass. Results of stepwise regression model to assess the effect of the most significant CWM traits and the most significant diversity indices (according to the previous multiple regression models) on biomass, in the lowland and the highland.
(PDF)

S5 Table Stepwise regression for the effect of CWM traits and diversity on change in vegetation composition. Results of stepwise regression model to assess the effect of the CWM traits and diversity indices on the change in vegetation composition (Bray-Curtis dissimilarity) in the lowland.
(PDF)
