# Supplementary material for: Functional Trait Changes, Productivity Shifts and Vegetation Stability in Mountain Grasslands during a Short-Term Warming
Source: PLoS One. 2015 Oct 29;10(10):e0141899. doi: 10.1371/journal.pone.0141899 (PMC4626038; doi:10.1371/journal.pone.0141899)
Supplement: S1 Table — Correlation coefficients between the CWM of traits: SLA, LDMC, height, start of first flowering, % prostrate, % rhizomes. (PDF) [file pone.0141899.s001.pdf]

S1 Table. Correlation coefficients between the CWM of traits: SLA, LDMC, height, start of first flowering, % prostrate, % rhizomes.

	SLA	LDMC	Start of first flowering	Height	% prostrate	% rhizomes
**SLA**	1.000	0.492	-0.627	0.007	-0.043	0.446
**LDMC**	0.492	1.000	-0.015	0.192	0.097	0.099
**Start of first flowering**	-0.627	-0.015	1.000	0.018	0.124	-0.365
**Height**	0.007	0.192	0.018	1.000	0.008	0.062
**% prostrate**	-0.043	0.097	0.124	0.008	1.000	-0.463
**% rhizomes**	0.446	0.099	-0.365	0.062	-0.463	1.000
