# Supplementary material for: Functional Trait Changes, Productivity Shifts and Vegetation Stability in Mountain Grasslands during a Short-Term Warming
Source: PLoS One. 2015 Oct 29;10(10):e0141899. doi: 10.1371/journal.pone.0141899 (PMC4626038; doi:10.1371/journal.pone.0141899)
Supplement: S2 Table — Correlation coefficients between the diversity indices: species richness SR, Simpson’s diversity index, and functional diversity FD. (PDF) [file pone.0141899.s002.pdf]

S2 Table. Correlation coefficients between the diversity indices: species richness SR, Simpson's diversity index, and functional diversity FD.

	SR	Simpson	FD
**SR**	1.000	0.864	0.298
**Simpson**	0.864	1.000	0.322
**FD**	0.298	0.322	1.000
