# Supplementary material for: Functional Trait Changes, Productivity Shifts and Vegetation Stability in Mountain Grasslands during a Short-Term Warming
Source: PLoS One. 2015 Oct 29;10(10):e0141899. doi: 10.1371/journal.pone.0141899 (PMC4626038; doi:10.1371/journal.pone.0141899)
Supplement: S3 Table — Results of multiple regression models to assess the effect of the initial plant functional traits (SLA, LDMC, height, start of first flowering, % prostrate, % rhizomes) at the beginning of the experiment on vegetation composition (Bray-Curtis dissimilarity), with transplant and site. See Fig 3 for a graphical representation. (PDF) [file pone.0141899.s003.pdf]

S3 Table. Results of multiple regression models to assess the effect of the initial plant functional traits (SLA, LDMC, height, start of first flowering, % prostrate, % rhizomes) at the beginning of the experiment on vegetation composition (Bray-Curtis dissimilarity), with transplant (highland and lowland) and site (p1 and p2 sites). See Fig. 3 for a graphical representation.

			Bray-Curtis			
	**Model**	**$R^2_{adj.}$**	**Estimate**	**Std. Error**	**t value**	***P***
**Site**	***[a]	0.625	-0.01	0.02	-0.74	0.464
**Transplant**			-0.54	0.21	-2.52	0.015
**Initial start of first flowering**			0.13	0.09	1.46	0.150
**Initial height**			-0.007	0.004	-1.67	0.101
**Initial SLA**			-0.02	0.02	-1.56	0.124
**Transplant: initial SLA**			0.02	0.01	1.81	**0.076**[b]

[a] ***P < 0.001

[b] P-values in bold indicate a tendency to a significant relationship
