# Supplementary material for: Functional Trait Changes, Productivity Shifts and Vegetation Stability in Mountain Grasslands during a Short-Term Warming
Source: PLoS One. 2015 Oct 29;10(10):e0141899. doi: 10.1371/journal.pone.0141899 (PMC4626038; doi:10.1371/journal.pone.0141899)
Supplement: S4 Table — Results of stepwise regression model to assess the effect of the most significant CWM traits and the most significant diversity indices (according to the previous multiple regression models) on biomass, in the lowland and the highland. (PDF) [file pone.0141899.s004.pdf]

S4 Table. Results of stepwise regression model to assess the effect of the most significant CWM traits and the most significant diversity indices (according to the previous multiple regression models) on biomass, in the lowland and the highland.

	Above-ground biomass					
	**Model**	**R^2 adj.**	**Estimate**	**Std. Error**	**t value**	***P***
Lowland						
**Intercept**	***[a]	0.87	-1256.96	796.88	-1.58	0.146
**Height**			44.85	8.37	5.36	**<0.001**[b]
**% rhizomes**			-1091.43	297.06	-3.67	**<0.01**
**FD**			972.13	540.35	1.80	0.102
Highland						
**Intercept**	(*)	0.19	119.10	36.41	3.27	**<0.01**
**% rhizomes**			136.38	67.66	2.02	0.067

[a] ***P < 0.001, (*) < 0.1

[b] P-values in bold indicate significant relationships.
