# Supplementary material for: Functional Trait Changes, Productivity Shifts and Vegetation Stability in Mountain Grasslands during a Short-Term Warming
Source: PLoS One. 2015 Oct 29;10(10):e0141899. doi: 10.1371/journal.pone.0141899 (PMC4626038; doi:10.1371/journal.pone.0141899)
Supplement: S5 Table — Results of stepwise regression model to assess the effect of the CWM traits and diversity indices on the change in vegetation composition (Bray-Curtis dissimilarity) in the lowland. (PDF) [file pone.0141899.s005.pdf]

S5 Table. Results of stepwise regression model to assess the effect of the CWM traits and diversity indices on the change in vegetation composition (Bray-Curtis dissimilarity) in the lowland.

	Bray-Curtis					
	**Model**	**R^2 adj.**	**Estimate**	**Std. Error**	**t value**	***P***
**Intercept**	*[a]	0.18	1.95	0.51	3.84	**<0.001**[b]
**Initial SLA**			-0.06	0.02	-2.78	**<0.01**
**Initial height**			-0.01	0.01	-1.56	0.130

[a] *P < 0.05

[b] P-values in bold indicate significant relationships
